# Online conspiracy communities are more resilient to deplatforming

**DOI:** 10.1093/pnasnexus/pgad324

**Published:** 2023-10-31

**Authors:** Corrado Monti, Matteo Cinelli, Carlo Valensise, Walter Quattrociocchi, Michele Starnini

**Affiliations:** CENTAI Institute, Corso Inghilterra 3, Torino (TO) 10138, Italy; Department of Computer Science, Sapienza University of Rome, Viale Regina Elena 295, Roma (RM) 00161, Italy; Centro Ricerche Enrico Fermi, Piazza del Viminale 1, Roma (RM) 00184, Italy; Department of Computer Science, Sapienza University of Rome, Viale Regina Elena 295, Roma (RM) 00161, Italy; CENTAI Institute, Corso Inghilterra 3, Torino (TO) 10138, Italy; Departament de Fisica, Universitat Politecnica de Catalunya, Campus Nord, Barcelona 08034, Spain

**Keywords:** deplatforming, content moderation, social networks, social media

## Abstract

Online social media foster the creation of active communities around shared narratives. Such communities may turn into incubators for conspiracy theories—some spreading violent messages that could sharpen the debate and potentially harm society. To face these phenomena, most social media platforms implemented moderation policies, ranging from posting warning labels up to deplatforming, i.e. permanently banning users. Assessing the effectiveness of content moderation is crucial for balancing societal safety while preserving the right to free speech. In this article, we compare the shift in behavior of users affected by the ban of two large communities on Reddit, *GreatAwakening* and *FatPeopleHate*, which were dedicated to spreading the QAnon conspiracy and body-shaming individuals, respectively. Following the ban, both communities partially migrated to Voat, an unmoderated Reddit clone. We estimate how many users migrate, finding that users in the conspiracy community are much more likely to leave Reddit altogether and join Voat. Then, we quantify the behavioral shift within Reddit and across Reddit and Voat by matching common users. While in general the activity of users is lower on the new platform, *GreatAwakening* users who decided to completely leave Reddit maintain a similar level of activity on Voat. Toxicity strongly increases on Voat in both communities. Finally, conspiracy users migrating from Reddit tend to recreate their previous social network on Voat. Our findings suggest that banning conspiracy communities hosting violent content should be carefully designed, as these communities may be more resilient to deplatforming.

**Significance Statement**

Addressing the effectiveness of deplatforming harmful online communities is crucial to strike a balance between societal safety and the right to free speech. We compare the effects of deplatforming a conspiratorial community that spreads misinformation with a nonconspiratorial one dedicated to generic hate speech. We quantify the behavioral changes of users who remain within the banning platform and those who migrate to a new, unmoderated one. The conspiracy community is more resilient to deplatforming, with more users fully migrating to the new platform, where they were able to recreate their previous interaction network.

## Introduction

Conspiracy theories posit that significant sociopolitical events are the result of a deliberate and coordinated scheme by a small group of influential individuals (1). While known historical conspiracies are limited in time, scope, number of actors, and complicacy, scholars distinguished a “conspiracy mindset” characterized by Hofstader’s paranoid style: the belief in “a vast, gigantic and yet subtle machinery of influence set in motion to undermine and destroy a way of life” (2, 3). The QAnon conspiracy theory is a prominent example: its adherents believe in a sinister global cabal consisting of powerful individuals who engage in cannibalism and pedophilia, and supposedly conspired against former U.S. President Donald Trump (4, 5). Emerged in 2017 as a grouping of far-right conspiracy theories, it developed a huge online following. Some of its believers have since been responsible of violent acts, harming those around them (6, 7). It has even been defined as an addiction-inducing cult, that can destroy one’s life (8). Online social media, where users can easily join communities around shared narratives (9–13), may form the ideal incubator for the growing of such conspiracy communities (14, 15). The social media platform Reddit, in particular, has been criticized for hosting extreme right-wing content (16), and possibly boosting violent and dangerous conspiracy theories from fringe websites into mainstream discourse (17, 18).

To contrast these harmful dynamics, as well as the diffusion of inappropriate content in general, most social media platforms implemented moderation policies, i.e. governance mechanisms that structure participation in a community to facilitate cooperation and prevent abuse (19). These policies differ in severity, being based on strategies such as post-warning labels, quarantines, shadow bans, the removal of posts, up to the permanent ban of single users or whole groups responsible for violating the platform’s usage policy. Warning labels are a nudging-like strategy used on several online social media to provide platform-mediated information to users about posts (20–22), which, however, may trigger a higher level of engagement (22, 23). Also quarantining, aimed at preventing direct access to and promotion of controversial communities, has been found to be essentially ineffective in terms of reduction of antisocial behaviors (24). *Deplatforming*, instead, is the attempt to limit the danger posed by an individual or a group by removing the platforms (e.g. specific channels, media, or websites) that they use to propagate their content (25). On Reddit, for instance, in 2015 five subreddits were closed due to violations of the anti-harassment policy, including r/fatpeoplehate—a large subreddit dedicated to weight-based bullying.

The efficacy of banning groups of users has been evaluated along two different, interdependent directions. On the one hand, community-level moderation can be effective *within* the affected platform. For instance, after the 2015 subreddit ban, users remaining on Reddit significantly reduced their level of hate speech (26). Also banning influential users who spread conspiracy and hate on Twitter has been shown to reduce their supporters’ activity and toxicity (27). On the other hand, users of banned communities can also decide to collectively migrate to alternative platforms: the efficacy of content moderation policies should thus be evaluated also by considering effects *between* platforms. This case was studied in terms of migration to websites, finding an overall reduction of activity despite increased toxicity (28), supporting the hypothesis that community bans can also be effective with respect to the broad web ecosystem. After Reddit’s 2015 ban, affected users massively joined Voat—a news aggregator website explicitly indicated as a safe harbor for communities banned from Reddit (29). Although the effects of community bans have been studied separately within or between platforms, a direct comparison between the behavior of users who migrate to the new platform and those that remain in the old one is still missing. Furthermore, since users who participate in conspiracy communities exhibit a higher engagement compared to other users (30, 31), such a comparison is crucial to understand the impact of deplatforming on this particular group of users.

To address the lack of research in this area, we quantitatively compare two groups of users affected by a community ban: users who migrated to a new platform and those who also remained on the old platform. We do so for two communities, the QAnonfocused subreddit *GreatAwakening*, banned in 2018, and the hate-speech-oriented subreddit *FatPeopleHate*, removed in 2015. Due to the lack of moderation, both communities identified Voat as their main resettlement platform. Our aim is to determine the impact of belonging to a conspiratorial community on user behavior with respect to deplatforming.

First, we estimate how many Reddit users decide to migrate to Voat and/or completely leave Reddit, following the ban. We show that the fraction of migrating users who also abandoned Reddit is much higher within the conspiracy-related community. Next, we analyze the behavior of migrating users by differentiating between those who stayed on the old platform and those who completely left it. Users who completely left Reddit and were part of a conspiracy community exhibit higher levels of activity on Voat compared to the nonconspiracy community. Toxicity strongly increases on Voat in both communities. Finally, we observe that the new conspirational community is also able to partially reproduce the old social network: users continue to interact with the same peers after migration, providing evidence for a more resilient community structure. Our findings show that deplatforming can effectively reduce the activity, size, and connections with other groups of both types of communities (32, 33). However, conspiracy communities tend to be more resilient, an element which should be taken into account when designing content moderation policies.

## Results

We analyzed the communities of *GreatAwakening* (GA) and *FatPeopleHate* (FPH) on Reddit and Voat, described in detail in Section Data. For the GA community, we considered the large QAnon-related subreddit r/GreatAwakening on Reddit and two QAnon-related subverses, v/GreatAwakening and v/theAwakening, on Voat. For the FPH community, we considered the subreddit r/fatpeoplehate on Reddit and the subverse v/fatpeoplehate on Voat. First, we estimate how many users join the new platform (Voat) and leave the old one (Reddit). Next, we compare the behavior of users between and within platforms, in terms of joined topical communities, activity, and toxic comments. Finally, we examine the resilience of the network structure of the two communities.

### Quantifying migration after deplatforming

Members of a banned community on a social media platform face two binary choices: (i) staying on the old platform or leaving and (ii) joining a new one to rebuild the banned community or not. The combinations of these choices define four possible behavioral classes: users (i) remaining on Reddit *and* joining Voat, (ii) remaining on Reddit *and* not joining Voat, (iii) leaving Reddit *and* joining Voat, and (iv) leaving Reddit *and* not joining Voat. Quantifying migrating users can be challenging, and it is often neglected. However, estimating the number of users for each behavioral class is crucial to understand the potential interest in a new environment and evaluating the impact of a ban on the broader web.

To this aim, we combine some measurements performed on the Reddit and Voat data sets with a minimal set of simplifying assumptions, which we describe in detail in the Methods section and briefly recap here. First, we identify the total number of users participating in the banned subreddits at the moment of the ban and quantify the fraction of these users who leave Reddit altogether after the ban. Then, we estimate the number of users joining Voat that might be users of the banned subreddits. Our estimation is based on two simplifying assumptions. First, we assume that new users joining a Voat community in the months following the ban of the corresponding subreddit (in excess with respect to the previous joining trend) are likely Reddit users fleeing the banned community. Second, we assume that users with the same username on Reddit and Voat correspond to the same individuals. Note that we do not assume that all users would adopt the same username on both platforms, just that a certain fraction of them does it. Under this assumption, two identical usernames correspond to the same individual, identifying a subset of the migrating users. Note that, except in the case of probabilistic matching based on username length (34), this assumption has been employed in other studies (28, 35), while more sophisticated user matching procedures (36) based on user profile details cannot be performed here due to the availability of usernames only. This assumption is also validated by our further analysis of their network, described later.

**Fig. 1.**
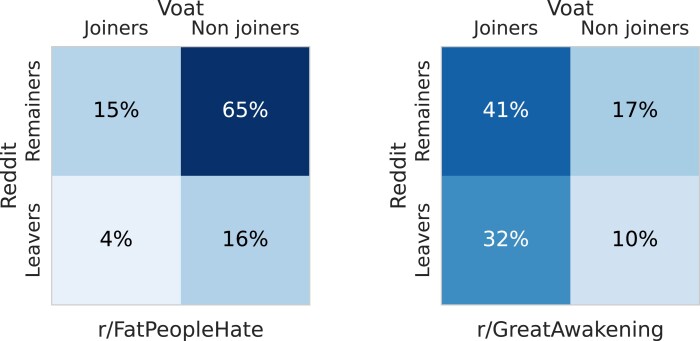
Estimated fraction of users in the four behavioral classes: Reddit remainers or leavers, and Voat joiners or nonjoiners. Users from *GreatAwakening* (right) are much more likely to completely leave Reddit and join Voat with respect to *FatPeopleHate* users (left).

These two assumptions allow us to estimate (see Methods) the number of users for each behavioral class, reported as a fraction of the total number of users of *GreatAwakening* and *FatPeopleHate* ($N_{\mathrm{GA}}=$ 24,569 and $N_{\mathrm{FPH}}=$ 70,739) in Fig. 1. We observe a considerable difference between GA and FPH in the fraction of users belonging to the four classes. First, only 20% of users (sum of the bottom row) leave Reddit after the FPH ban, while more than 40% do the same after the GA ban. More specifically, only 4% of users fully migrate from Reddit to Voat after the FPH ban, while 32% do so for GA. These users are the most attached to the community banned. Conversely, users who continue to use Reddit without joining Voat after the ban are many more for FPH than for GA (65% vs 17%).

These findings suggest that FPH users may perceive their participation to the community as a casual activity, and not as a part of their identity. On the contrary, users populating the GA community are much more involved in the subreddit and react very differently to the community ban. Overall, the deplatforming seems to affect the behavior of a part of users, but the fraction of the users affected is determined by the importance of the banned subreddit for the users involved.

Of course, the specific numbers we find depend on how “participation” is defined: for instance, we can consider users who post at least *n* messages in the given community. This way, we would subselect users who are more active and thus are more likely to appear in the Voat joiners class. However, we find that the striking difference we observe between the two communities is robust to this change, hinting at a general behavioral difference between the two groups.

### Migrating users change their behavior

We now focus on the behavior of the users joining Voat. Since we want to compare their behavior on Voat and Reddit, we only consider users with the same username on both platforms, which we assume to belong to the same individual, as described in the previous section. As detailed in Methods, this focus group is composed of 1,341 users for *GreatAwakening* and 2,030 for *FatPeopleHate* who use the same pseudonyms on both platforms. For these users, we are able to gather their entire posting history on Reddit and on Voat.

In this section, we show how the interest in different communities by this subgroup of users changes *within* and *between* platforms (Reddit and Voat). Then, we investigate whether users leaving Reddit altogether show any difference in behavior with respect to users who keep using both platforms. To do so, we compare their behavior on Reddit and Voat in the 6 months before or after the ban, respectively, in terms of participation in other communities, activity, and usage of toxic language.

#### Community participation

**Fig. 2.**
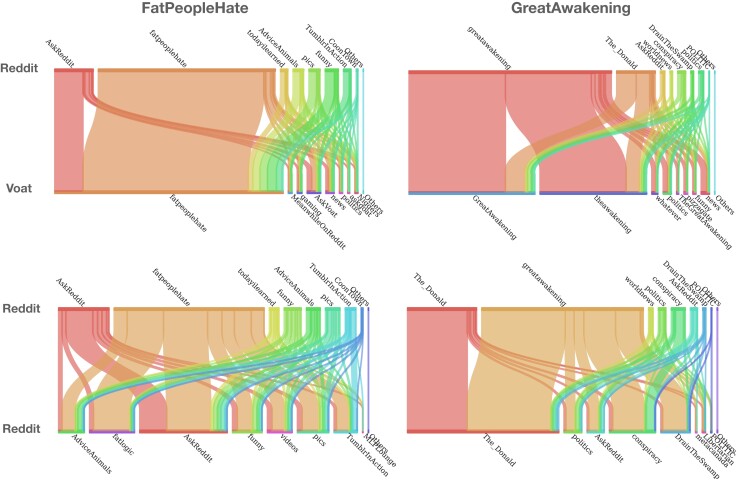
Participation of users in Reddit and Voat communities. The top row shows users migrating from Reddit communities, joined up to 6 months before the ban date (on the top side of the plot) to Voat communities joined up to 6 months after the ban date (bottom side). The bottom row shows users remaining on Reddit, communities joined up to 6 months before the ban (top side of the plot) versus communities joined up to 6 months after the ban (bottom side). *FatPeopleHate* is on the left column and *GreatAwakening* on the right. In each plot, the width of each community is proportional to the number of users participating in that community, weighted by the number of messages posted there. Disclosure: This figure contains offensive language, which neither NAS nor the authors condone. Language is included as it appeared in the Reddit and Voat forums used in the research project.

We start by studying how community participation changes for users migrating from Reddit to Voat. Figure 2 (top row) shows the participation of users in the new communities on Voat with respect to the old ones joined on Reddit, for *FatPeopleHate* (left) and *GreatAwakening* (right). In these Sankey plots, the width of each Voat or Reddit community (on the top and bottom of the plot, respectively) indicates the number of users participating in that community in the 6 months before the ban (for Reddit communities) or after it (for Voat). All users have the same weight (set equal to 1), split proportionally to the number of messages posted in the communities they participate in.

We observe that users tend to mainly join new communities on Voat corresponding to the old, banned ones. For instance, FPH users on Reddit mainly join the new FPH community on Voat, and the same is true for GA users. However, while FPH users join also generalist communities on Voat, such as gaming and technology, GA users tend to choose communities related to conspiracy: they mainly join v/GreatAwakening and v/theAwakening, many also post on v/Conspiracy and v/pizzagate.

Then, we perform the same analysis *within* Reddit. Figure 2 (bottom) shows how participation to communities on Reddit shifts to other Reddit communities after the ban, for users remaining in Reddit. Users in the conspiracy community massively move to r/The_Donald (70% of users do so) and r/conspiracy (38%), as well as r/DrainTheSwamp (29%), another trumpist and conservative subreddit (37). On the contrary, only 6% of users from FPH participate in a community with a topic similar to the former one (r/fatlogic) after the ban. Note, however, that FPH users tried to create more than 99 subreddits related to the same theme, which were also quickly banned by Reddit (38).

#### Activity

**Fig. 3.**
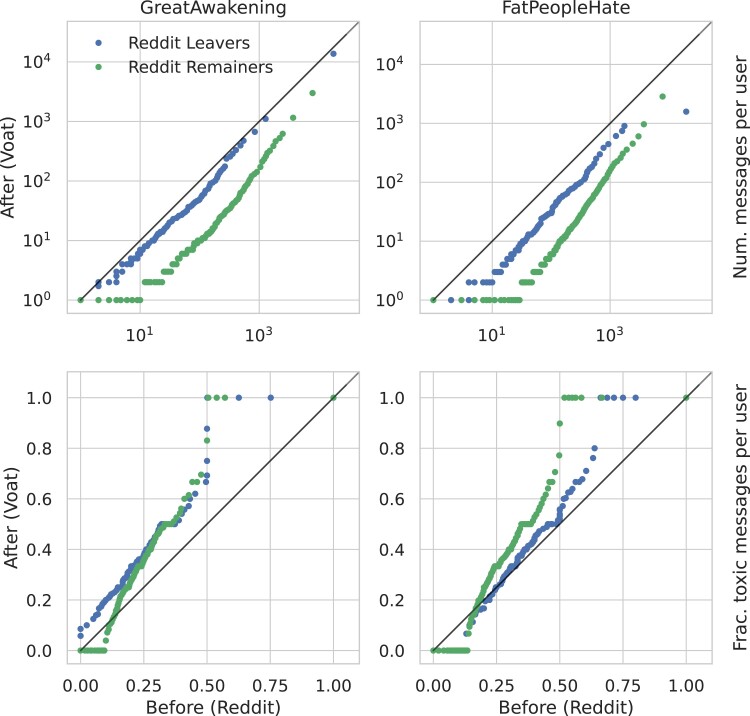
Activity and toxicity on Reddit and Voat. QQ Plot comparing the distribution of the number of messages (top row) and fraction of toxic messages (bottom row) per user on GA (left column) and FPH (right column), in the 6 months before the ban on Reddit (*x*-axis) and 6 months after the ban on VOAT (*y*-axis). We distinguish between users leaving (blue dots) and remaining (green dots) on Reddit. Only Reddit leavers of the GA community maintain a level of activity comparable to the old platform, while the toxicity of the messages increases upon migration for all classes of users.

Then, we compare the number of messages posted on Reddit (pre-ban) and on Voat (post-ban). Within this comparison, we differentiate between two groups of migrating users: those who keep participating on both platforms and those who leave Reddit. Figure 3 (top row) shows results for GA and FPH as a quantile–quantile (QQ) comparison: if two distributions are similar the quantiles will lie on the diagonal; dots below the diagonal indicate a decrease of the quantity on the *y*-axis with respect to that on the *x*-axis, dots above indicate an increase. Figure 3 (top row) shows that the activity on the new platform is generally lower than on the old one, for both very active and less active users. This observation holds for both communities, *GreatAwakening* (left column) and *FatPeopleHate* (right column). One possible explanation is that since the new-borne community is still in its infancy, it generates less engagement. This observation suggests that, to some extent, deplatforming is effective in reducing engagement in both communities.

However, Fig. 3 (top row) also shows that Reddit leavers (blue dots) are more active on the new Voat communities than users remaining on Reddit (green dots), for both GA and FPH. Users who completely leave the old platform are therefore relatively more engaged with the new platform. *GreatAwakening* users remaining on Reddit post on average 13.3 messages per month, compared to 16.1 for leavers. The difference between leavers and remainers is slightly less prominent for *FatPeopleHate*: the average number of messages per month is 11.6 for remainers and 12.8 for leavers (note that we use a log scale in Fig. 3). In order to assess whether remainers and leavers are indeed significantly different in terms of change in activity, we perform a two-sided Mann–Whitney *U* statistical test, to compare the ratio of messages between the new and the old platform for leavers and remainers. This test shows that the two groups are very different for both GA and FPH (*U* statistics greater than $10^{5}$, *p*-value $<10^{-25}$).

Furthermore, one can see that *GreatAwakening* users leaving Reddit show a level of activity on Voat very similar to Reddit’s one (blue dots almost on the diagonal in the left panel of Fig. 3). Conversely, activity of *FatPeopleHate* users on Voat leaving Reddit is considerably lower compared to Reddit. This difference between *GreatAwakening* and *FatPeopleHate* users is confirmed by the two-sided Mann–Whitney *U* statistical test, comparing activity on Reddit and Voat for users leaving Reddit. The test indicates a *U* statistic for *FatPeopleHate* users of $5.4\cdot 10^{4}$, *p*-value $<10^{-14}$, while the activity difference between Reddit and Voat for *GreatAwakening* users is still significant but much less so (*p*-value <0.0002). Therefore, the growth of the new GA community on Voat is mainly driven by users fully migrating to the new platform, while this effect is less evident on FPH.

#### Toxicity

Next, we compare the use of toxic language between platforms, again distinguishing Reddit leavers and remainers. Quantifying toxic messages is relevant to assess whether a less regulated environment induces an increase in the use of toxic speech. To this aim, we classify each message with the IMSyPP classifier (39) (see Methods). Figure 3 (bottom row) shows a QQ comparison of the fraction of toxic messages between platforms. We observe that the level of toxicity is generally much higher on the new Voat communities than on the corresponding banned subreddits. In particular, the average fraction of toxic comments increases by 9 percentage points for GA (from 21% to 30%) and by 7 points for FPH (from 29% to 36%). In both cases, the difference between the two groups is significant according to a two-sided Mann–Whitney *U* statistical test (*p*-value $<10^{-15})$. The figure shows a more pronounced increase for users with the largest fraction of toxic messages, on both GA and FPH communities. Therefore, it appears that while deplatforming can partially reduce the activity of users on the new platform, it also strongly increases their toxicity, for both conspiracy and nonconspiracy communities.

**Fig. 4.**
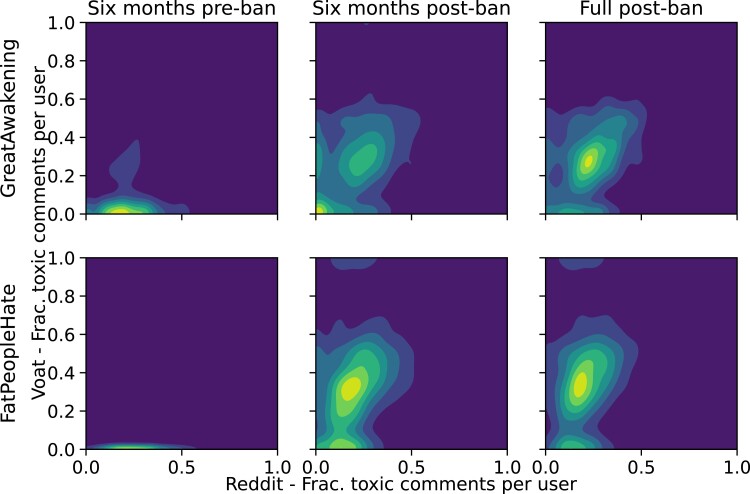
Temporal evolution of the levels of toxic speech on Reddit (*x*-axis) vs Voat (*y*-axis), on *GreatAwakening* (top row) and *FatPeopleHate* (bottom row). We distinguish between the fraction of toxic messages on Voat 6 months before the ban of the subreddit (left column), 6 months after it (middle), and up to 2 years after it (right column). The fraction of toxic comments increases in both communities on the new platform, persisting up to 2 years after the ban date.

We investigate this phenomenon in more detail by disentangling the temporal evolution of the toxic speech levels. Our aim is to understand if the toxicity increases on Voat after the migration of users from Reddit and if such an increases is merely temporary or if it persists at elevated levels for more than 6 months after the ban date. Figure 4 shows the density of toxic messages in the Reddit vs Voat phase diagram, for GA (top row) and FPH (bottom row). We distinguish between messages on Voat 6 months before the ban date (left column), 6 months after the ban (middle), and up to 2 years after the ban (right column). This figure shows that as soon as the new platform gets populated, the number of toxic comments on the new Voat community increases with respect to the banned subreddit. In more detail, the majority of users posted about 20% of toxic messages on *GreatAwakening* on Reddit (slightly less on *FatPeopleHate*), while they post almost 40% of toxic messages on Voat. Toxicity levels exhibit a noticeable increase even beyond the 6-month mark following the ban date.

### Measuring community resilience to bans

**Fig. 5.**
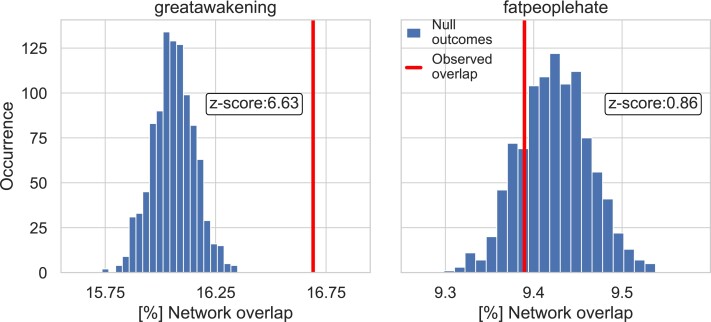
Network overlap in the r/greatawakening (left) and r/fatpeoplehate (right) communities (vertical line), compared with a null model reshuffling the interactions (histogram). In *GreatAwakening*, the observed overlap is not compatible with random fluctuations. Conversely, *FatPeopleHate* shows an overlap fully compatible with random re-organization.

The results displayed so far suggest a difference between the users participating to FPH and GA in terms of their response to the community ban that could be reconducted to a different sense of community belonging. We further test this observation by means of a network-based analysis, i.e. we compare the two social networks on the banned subreddits and on the corresponding communities on Voat, where two users are connected if they commented on the same post (see Methods). As we are considering users that appear on both social media platforms, we can investigate the overlap in the structure of the two networks, that is the social relations of the banned community that are preserved after the migration to Voat. Note that so far we considered users from two QAnonrelated communities on Voat, v/GreatAwakening and v/theAwakening. Since we are directly comparing social networks, in the following we focus only on the largest of the two communities, i.e. we compare r/GreatAwakening to v/GreatAwakening, discarding users from v/theAwakening. We find that 16.7% of the GA banned community is reconstructed on Voat, while this fraction is 9.4% for FPH. We test the significance of these values by building a null model which shuffles the set of posts commented by each user, see Methods. Figure 5 shows that the empirical overlap for GA is not compatible with the null model (z-score: 6.6), while it is for FPH (z-score: 0.86).

First, this finding consolidates our assumption that users with the same username on both platforms correspond to the same individual, since these results cannot be attributed to chance. Then, this finding confirms that the conspiracy community is more resilient to deplatforming: the social engagement created by sharing the same narrative helps conspiracy users to reconstruct even the user–user interactions of the banned community. A possible mechanism that could explain this effect is that the broad narrative behind the GA conspiracy theory is tied to the study and discussion of a number of sub-issues and topics. Indeed, sub-communities of users might gather around such topics, leading to a resilient community structure in *GreatAwakening*. Conversely, the observed overlap is fully compatible with random fluctuations.

## Discussion

In this article, we investigated the difference in the behavior of participants of deplatformed subreddits, comparing a conspiracy and a nonconspiracy community. The conspiracy narrative is one of the most recognizable aspects characterizing online discussions (31) and—despite the number of conspiracy posts being negligible with respect to other categories, (e.g. news (11, 40))—conspiracy still finds room for active online debates. We find that users in the conspiracy community are much more likely to leave the moderated platform altogether and join a new, unmoderated one. Furthermore, while both communities display a decrease in activity, the decrease is less prominent for the conspiracy community. Users migrating from the deplatformed conspiracy community are more likely to recreate their social ties on the new platform.

Overall, our findings point to a higher resilience—i.e. the ability to recreate the old community after a disruption—of conspiracy groups, that may be due to a different involvement with the narratives that define the two groups. Since conspiracy narratives strongly affect the sense of self of their participants (8), they are likely to develop a strong attachment to the community, to its norms, narratives, and values, with respect to users casually participating in a hate speech community. Moreover, an effort in preserving this set of common values and knowledge might explain their ability in reconstructing social ties. Our findings highlight new aspects of participation in conspiracy communities, in line with other results regarding conspiracy theories on Reddit about the mechanisms that drive users to join (14), participate (41), and leave conspiracy communities (15). It is important to remark that not only GA users but also the FPH community shows some level of shared knowledge among their participants, with many conversations related to scientific or pseudo-scientific proofs that obesity is detrimental to one’s health. Likewise, they used a lot of slurs towards overweight people, which may indicate the rise of a linguistic component within the community (42). Therefore, the greater resilience of the conspiracy community may have meaningful implications, suggesting stronger social ties among its members, potentially driven by a broader shared knowledge and value system.

More work is needed to carefully evaluate these aspects highlighted by our work, and to overcome its limitations. For instance, our method for finding common users across platforms—i.e. simply matching their usernames—could be improved by adopting more complex techniques, such as text mining and author detection. We remark that we analyzed only the behavioral shift of this subgroup of users, while we cannot assume that they are representative of the general population of their community. Another limitation resides in comparing two communities banned with a considerable time difference. Indeed, r/FatPeopleHate was banned in 2015, when Voat was a very small platform without an active community, while when r/GreatAwakening was banned in 2018, several QAnonrelated communities existed already on Voat. The user base in Voat considerably grew between 2015 and 2018, thus possibly explaining the lower activity of v/FatPeopleHate users with respect to v/GreatAwakening and v/theAwakening users. Furthermore, our analysis considers only two communities: while data availability constrained this choice, other comparisons of conspiracy vs nonconspiracy communities may be undertaken in the future. As a consequence, we do not know how well our findings generalize to other de-platformed communities—more research is needed in this direction. For instance, migration to other platforms besides Voat could be taken into account, by following our framework. Moreover, our study is purely observational: a causal analysis of the relationship between user behavior, participation in conspiracy communities, and deplatforming would be needed. However, our work is a necessary first step, as it shows that such a link could provide valuable insights into the effect of moderation policies on conspiracy communities. Finally, a more in-depth investigation of the preservation of norms, values, and narrations during the migration could further corroborate our hypothesis.

Our findings have implications combining both technical and social aspects. Social media platforms must weigh in the effectiveness of severe moderation with the potential social costs it brings, such as migration to alternative platforms and heightened user toxicity. With indications that conspiracy thinking is rising, possibly also due to growing economic disparities (43), social media platforms should thoroughly evaluate counter-measures.

## Data and methods

In this section, we describe in detail the data set used and the methodology to estimate the number of users migrating from Reddit to Voat.

### Data

Here, we provide an overview of our two main data sources: Reddit and Voat, and in particular the two communities we analyze, *GreatAwakening* and *FatPeopleHate*.

#### Reddit

Reddit is a social content aggregation website, organized in topical communities, called *subreddits*, centered around a variety of topics where all users must have a pseudonymous account in order to participate. Users can post submissions in these subreddits, and comment on other submissions and comments, thus creating a tree structure for the overall discussion. We call a message a generic piece of user-generated content, when the distinction between submission and comment is not relevant. In addition, users can also upvote a submission to show approval, appreciation, or agreement (and their opposites with a downvote). The score of a submission is the number of positive votes minus the number of negative votes it has received. Differently from other social media like Facebook or Twitter, Reddit’s homepage is organized around subreddits, and not on user-to-user relationships. As such, the subreddits chosen by a user represent the main source of the information they consume on the website.

Reddit has already been studied within a rich set of research frameworks. For instance, the study of user engagement and interactions between highly related communities (44, 45), the analysis of post-election political analyses (46), or the impact of linguistic differences in news titles (47). Interestingly, it has already been used to explore harmful social dynamics on online social media such as hate speech (42) or cyberbullying (48). Health-related issues have also been studied on Reddit, like mental illness (49), as well as the opioid epidemics in the United States (50, 51). We collect public data from Reddit using the Pushift collection (52).

#### Voat

Voat.co was a news aggregator website, shut down on December 25, 2020, that has been indicated as a safe harbor for communities banned from Reddit (29). Like Reddit, discussions on Voat occurred in specific groups of interests called “subverses.” Voat has been also defined as a “clone” of Reddit,^a^ since it mimicked its functionalities and interface: users can subscribe to subverses of interest, comment, upvote, and downvote the comments and submissions in a tree-like commenting system. In this article, we use the dataset collected by (29).

#### GreatAwakening

Among the banned communities on Reddit that reportedly migrated on Voat, an example of particular interest is r/GreatAwakening, a subreddit dedicated to the diffusion of the QAnon conspiracy theory. This subreddit has been identified as the largest QAnonrelated community on Reddit (53): it had an active user base with over 71,000 subscribers and an average of 10,000 comments per day, and it was banned on the September 18, 2018 for repeated content violations. On Voat, we consider two large QAnonrelated subverses: v/GreatAwakening and v/theAwakening, two of the most engaged communities (54).

#### FatPeopleHate

In order to study the difference in the effects of deplatforming between a conspiracy-related group, *GreatAwakening*, and other types of communities, we consider r/FatPeopleHate, a subreddit banned for hate speech, as a comparison. While many other communities have been banned over the years, this subreddit is the only one that is, at the same time, nonrelated to conspiracies, and for which anonymized data is available for both Reddit and Voat. r/FatPeopleHate was a subreddit entirely dedicated to body-shaming individuals, by posting pictures of overweight people that were then ridiculed.^b^ The subreddit internal rules prohibited users from expressing “fat sympathy.”^c^ On June 6 2015, the subreddit was banned: with over 150,000 subscribers at the time of the ban, it has been one of the largest banned subreddits. As in the case of *GreatAwakening*, previous work reported that part of *FatPeopleHate* users migrated to Voat (29), with the growth of an analogous subvoat with the same name after the Reddit ban.

### Methods

#### Estimate the number of migrating users

The ban of a community by a social media platform can be answered by its members with two binary choices: they can decide to (i) remain or leave the old platform and (ii) join or not a new one to reconstruct the community banned. We indicate by $U_{s}$ the set of all users participating in the banned subreddit *s* at the moment of the ban, with cardinality $N_{s}$. We indicate by $R_{s}$ the subset of $U_{s}$ formed by users who keep participating in Reddit after the ban date, with cardinality $R_{s}$. Conversely, users who leave Reddit after the ban are the complement set of $R_{s}$, i.e. ${\bar{R}}_{s}=U_{s}\setminus R_{s}$, with cardinality $|{\bar{R}}_{s}|=N_{s}-R_{s}$. In the same way, we indicate by $V_{s}$ the subset of $U_{s}$ formed by users who join Voat after the ban date ($|V_{s}|=V_{s}$), and by ${\bar{V}}_{s}=U_{s}\setminus V_{s}$ the complement set of users not joining Voat ($|{\bar{V}}_{s}|=N_{s}-V_{s}$).

The four possible behavioral classes are defined as the intersection of these sets: users (i) remaining on Reddit *and* joining Voat, R∩V, (ii) remaining on Reddit *and* not joining Voat, $R\cap \bar{V}$, (iii) leaving Reddit *and* joining Voat, $\bar{R}\cap V$, and (iv) leaving Reddit *and* not joining Voat, $\bar{R}\cap \bar{V}$. Our first goal is to provide a tentative estimate for the number of users for each of these four behavioral classes. Indeed, quantifying migrating users is a generally overlooked task, that can provide us estimates of the potential interest towards a new environment and, in turn, the effectiveness of the ban with respect to the broad web environment. To this aim, we combine some measurements performed on the Reddit and Voat data sets with a minimal set of simplifying assumptions.

First, we identify the total number of users participating in the banned subreddit at the moment of the ban by counting users posting at least one submission or comment in the previous 6 months. This count gives us $N_{\mathrm{GA}}=$ 24,569 user for *GreatAwakening* and $N_{\mathrm{FPH}}=$ 70,739 users for *FatPeopleHate*. Then, we detect how many users in this set are still active on Reddit in the 6 months following the ban date of each community, over the whole Reddit, to quantify the users leaving Reddit altogether. This gives us the number of Reddit remainers, i.e. users that show nonzero activity (as submission or comment) in any subreddit after the ban. We obtain $R_{\mathrm{GA}}=$ 14,243 for *GreatAwakening* and $R_{\mathrm{FPH}}=$ 56,918 for *FatPeopleHate*.

**Fig. 6.**
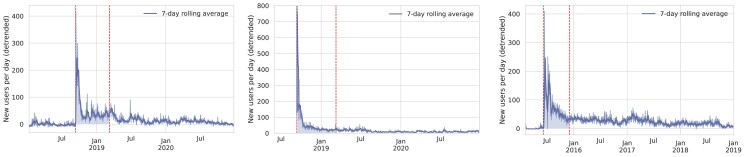
Number of new users per day in the Voat communities of v/GreatAwakening (left), v/theAwakening (middle), and v/FatPeopleHate (right) after detrending (i.e. removing the average pre-ban number of new users per day). Note that the v/theAwakening community was created the same week of the ban date, so we did not detrend the number of new users. In each plot, the first vertical dashed line indicates the date of the ban of the corresponding Reddit community and the second one corresponds to 6 months after such ban date.

Next, we wish to estimate the number of users joining Voat that might be users of the banned subreddits. We recall that we consider one subverse for *FatPeopleHate*, v/FatPeopleHate, and two QAnonrelated subverses, namely v/GreatAwakening and v/theAwakening. To do so, first, we count the number of daily new users in the GA and FPH communities on Voat, then, we detrend this quantity by subtracting the average pre-ban number of daily new users. We report the three time series in Fig. 6. This plot shows a large spike around the respective Reddit ban date of each community, suggesting a prominent effect of the Reddit ban on Voat. After the spike, the influx of new users rapidly decreases but remains higher with respect to the baseline for some time. Consequently, one can assume that users joining Voat mostly come from banned subreddits, at least for some time after the ban date. While it is not possible to precisely estimate when the effect of the Reddit ban ends, one can see that the number of daily new users on FPH became steady 6 months after the Reddit ban (Fig. 6). By following these observations, we assume that the number of (excess) new users joining a Voat community in the months following the ban of the corresponding subreddit are most likely Reddit users fleeing the banned community. This is our first simplifying assumption. To meaningfully compare the two communities, we choose 6 months as the defining interval for both groups. We discuss the robustness of this assumption below. Therefore, we interpret the shaded area in Fig. 6 as the number of Reddit users migrating on Voat, obtaining $V_{\mathrm{GA}}=$ 17,855 and $V_{\mathrm{FPH}}=$13,342. For $V_{\mathrm{GA}}$, we consider the union of the two sets of 12,049 users joining v/GreatAwakening and 9,844 users joining v/theAwakening. The fact that the union is much smaller than the sum indicates that many users joining the first community also joined the second.

At this point, for each subreddit *s*, we know how many users remain on Reddit ($|R_{s}|=R_{s}$), or leave it altogether ($|{\bar{R}}_{s}|=N_{s}-R_{s}$), join Voat ($|V_{s}|=V_{s}$) or not ($|{\bar{V}}_{s}|=N_{s}-V_{s}$). The only missing information to estimate the size of the four behavioral classes is how many users remain on Reddit *and* join Voat, i.e. the cardinality of the intersection R∩V. The other classes’ sizes can be easily derived by subtraction, as detailed later.

To this aim, we consider a subset of the migrating users, namely users who appear with the same username on a subreddit and, after the ban date, on the corresponding community on Voat. These users are $n_{\text{GA}}=1,341$ and $n_{\text{FPH}}=2,030$. Our second simplifying assumption is that these users with the same username correspond to the same individuals. While we cannot be sure that the behavior of this subset is representative of the general population of their respective community, we checked that their activity, toxicity, and score (upvotes and downvotes of their comments) are qualitatively similar to a random sample of users from the same subreddit. We thus calculate the fraction of users in the subsets $n_{\text{GA}}$ and $n_{\text{FPH}}$ who also belong to the set R, i.e. who are still active on Reddit, as users posting on any other subreddit after the ban of their community. These fractions read $\rho_{\mathrm{GA}}=0.59$ and $\rho_{\mathrm{FPH}}=0.80$: the difference in these numbers is the first suggestion that the two communities show a fundamental difference in the behavioral response to their deplatforming. By assuming that these fractions are the same for all users remaining on Reddit and joining Voat, the number of users in this class is simply $|R_{s}\cap V_{s}|=\rho_{s}\cdot V_{s}$.

As a further robustness check, we checked that these fractions do not depend on the activity of users, i.e. the fraction of users still active on Reddit is the same regardless of their activity. Conversely, the number of users abandoning Reddit in favor of Voat will be $|{\bar{R}}_{s}\cap V_{s}|=(1-\rho_{s})\cdot V_{s}$. The cardinality of the two last sets (users not joining Voat) is obtained by subtracting these numbers from the respective totals. The number of users remaining on Reddit and not joining Voat is $\left|R_{s}\cap {\bar{V}}_{s}|=R_{s}-(\rho_{s}V_{s}\right)$, while the number of users leaving Reddit and not joining Voat is $|{\bar{R}}_{s}\cap {\bar{V}}_{s}|=N_{s}-R_{s}-(1-\rho_{s})V_{s}$.

**Fig. 7.**
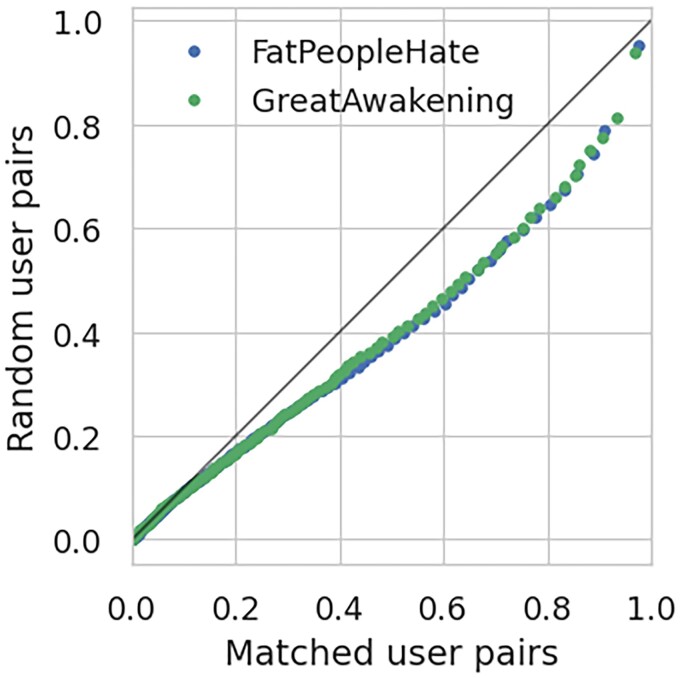
QQ Plot comparing the distributions of the cosine similarity of messages authored by matched users on Reddit and Voat. We compare the similarity between matched user pairs (*x*-axis) and random user pairs within the same set (*y*-axis), for both *FatPeopleHate* and *GreatAwakening*. The similarity of messages between Reddit and Voat by matched user pairs is clearly higher than the one between random user pairs.

Finally, we validate that the matched users are likely to correspond to the same individuals, by performing the following test. For each of the two communities (GA and FPH), we considered all the comments made by the users matched with common usernames before the ban date on Reddit (subreddits r/GreatAwakening and r/FatPeopleHate) and after the ban on Voat (subverses v/GreatAwakening and v/theAwakening for GA, v/FatPeopleHate for FPH). Then, we concatenate all the comments of the same user, obtaining two documents for each matched user for each community, one corresponding to comments on Reddit and the other corresponding to comments on Voat. Next, we check that these two documents are likely to be authored by the same user. To this aim, we compute the cosine similarity between the TF-IDF representations of the documents of two groups of paired users: matched users and random pairs from the same set. We then compare the distribution of these similarities. Figure 7 shows a QQ comparison between the two similarity distributions (matched user pairs vs random user pairs), confirming that they are different: messages authored by matched users are much more likely to be similar, for both communities. A KS test confirms that the two similarity distributions (matched user pairs and random pairs) are indeed different, with $p<10^{-5}$ for both communities.

#### Toxic comments detection

In order to detect toxic comments, we used the IMSyPP classifier (39) (publicly available^d^) to label each comment on Reddit and Voat as toxic or not. We define a message as toxic if it is classified into one of the following categories: (i) *inappropriate*, the message contains terms that are obscene or vulgar, but the text is not directed to any person or group specifically; (ii) *offensive*, the comment includes offensive generalization, contempt, dehumanization, or indirect offensive remarks; or (iii) *violent*, the comment’s author threatens, indulges, desires or calls for physical violence against a target; it also includes calling for, denying or glorifying war crimes and crimes against humanity. If the message is classified in the remaining category, namely *appropriate*, it is considered nontoxic. The accuracy for toxicity classification of the IMSyPP classifier is 0.84 (39). As a test of robustness, we contrast the results of this classifier with those of the Google Perspective API, whose toxicity score is in [0,1]. Results are in good agreement, obtaining a ROC AUC between the two classifiers of 0.94.

#### Social network analysis

We start by reconstructing a bipartite network, linking posts *p* and users *u* that commented on them, separately on each platform: we denote these networks as $B_{R}(p,u)$ on Reddit, and $B_{V}(p,u)$ on Voat. Next, we consider the unimodal projection on the user side of each bipartite network: we obtain two networks of users where users *u* and *v* are connected if and only if they commented on the same post, on the banned subreddit or on the corresponding community on Voat. We indicate these two user networks as $G_{R}=(N_{R},E_{R})$ and $G_{V}=(N_{V},E_{V})$, respectively. As we are considering users that appear on both social networks, we can investigate the overlap in the structure of the two networks. We define the overlap in the structure of the two networks as the fraction of edges of the Reddit network that are present also in the Voat one, that is $\frac{\left|E_{R}\cap E_{V}\right|}{\left|E_{R}\right|}$. We obtain that 16.7% of the GA banned community is reconstructed on Voat, while this fraction is 9.4% for FPH.

To assess the significance of these values, we built a null model that randomizes the Voat bipartite network $B_{V}(p,u)$, by shuffling the set of posts commented by each user thus creating a new bipartite network called $B_{V}^{\prime }(p,u)$. Afterward, we perform the projection of the randomized bipartite network obtaining $G_{V}^{\prime }(p,u)$, and we compute the overlap with the projection obtained by the empirical bipartite network obtained from Reddit $G_{R}(p,u)$. Note that the degrees of the nodes are preserved in the randomization. We repeat the randomization process 10,000 times and we obtain a Gaussian-like distribution of null overlap values.

## Data Availability

The data and code to reproduce results of this article are publicly available at https://github.com/corradomonti/deplatform-resilience.

## Footnotes

a The Guardian, *“Reddit clone Voat dropped by web host for ‘politically incorrect’ content.”*

b The Verge, *“Reddit bans ’Fat People Hate’ and other subreddits under new harassment rules.”*

c Washington Post, *“These are the 5 subreddits Reddit banned under its game-changing anti-harassment policy—and why it banned them”*.

d
https://huggingface.co/IMSyPP
